# Effect of Stem Design and Positioning on the Leg Axis after Total Hip Arthroplasty: A Secondary Analysis

**DOI:** 10.3390/jcm13154453

**Published:** 2024-07-30

**Authors:** Benjamin Fey, Marco Brenneis, Felix Stief, Stefan van Drongelen

**Affiliations:** 1 Department of Trauma Surgery and Orthopedics, University Hospital Frankfurt, Goethe University Frankfurt, Marienburgstr. 2, 60528 Frankfurt, Germany; benjamfey@gmail.com (B.F.); brenneis@med.uni-frankfurt.de (M.B.); felix.stief@bgu-frankfurt.de (F.S.); 2 Dr. Rolf M. Schwiete Research Unit for Osteoarthritis, Department of Trauma Surgery and Orthopedics, University Hospital Frankfurt, Goethe University Frankfurt, Marienburgstr. 2, 60528 Frankfurt, Germany

**Keywords:** total hip arthroplasty, leg alignment, stem design, hip osteoarthritis, femoral offset

## Abstract

**Background/Objectives:** Various parameters, like femoral offset and leg length, are associated with good patient outcomes after total hip arthroplasty. In this prospective study, the effects of stem design, its placement in the proximal femur and the resulting femoral offset on the total leg axis were investigated. **Methods:** The 27 patients included in this study received biplanar radiography (EOS^®^, EOS Imaging) with 3D reconstruction using sterEOS^®^ both preoperatively and postoperatively. For all leg alignment parameters obtained from the 3D reconstruction and from measurements using mediCAD, the deltas between the postoperative and preoperative values were determined. Patients were divided into those who received a short-stem prosthesis and those who received a straight-stem prosthesis. **Results:** The change in femoral offset with the implantation of a short-stem prosthesis was significantly greater than that with the implantation of a straight-stem prosthesis (11.4 ± 5.9 vs. 4.6 ± 7.4 mm, *p* = 0.014). Compared with the straight-stem implantation, short-stem implantation caused a significantly greater increase in the varus orientation of the leg (−1.4 ± 0.9 vs. −0.4 ± 1.4°, *p* = 0.048). There was no significant difference in the positioning of the short-stem prosthesis compared to the straight-stem prosthesis in the proximal femur (3.6 ± 3.1 vs. 2.6 ± 1.9°, *p* = 0.317). **Conclusions:** These findings substantiate the impact of prosthesis design on offset and leg alignment. The implantation of short-stems is more variable and requires precise planning. Intraoperative non-physiological offset changes and varus deviation of the leg axis should be avoided. Trial registration: This study was retrospectively registered with the German Clinical Trials Register (DRKS) under the number DRKS00015053 on the 1 August 2018.

## 1. Introduction

Since 1938, prosthetic components have been further developed and the cementless fixation technique has become increasingly established. The Zweymüller straight-stem prosthesis from 1980 is still in use today and has shown very good long-term results [1]. In recent years, short-stem prostheses have been developed that utilize the principle of proximal force application and allow for soft-tissue-sparing surgery with a smaller prosthesis size [2]. The service life of standard total hip arthroplasty (THA) has been steadily improved by further development of the implants and optimization of the surgical procedure. The international prosthesis registry data show that after complication-free implantation of a modern prosthesis, the 10-year survival rate of the implanted prosthesis can reach more than 90% [3,4]. In addition to the patient’s own risk factors, a favorable postoperative outcome and the occurrence of complications are primarily influenced by exact preoperative planning and correct surgical execution of the procedure [5].

Various parameters for achieving a favorable outcome after THA have been established in the past to ensure optimal postoperative function [6]. One important parameter for the function of the artificial hip joint is the offset. A satisfactory gait pattern can only be achieved with adequate offset reconstruction and the resulting functionality of the abduction muscles [7,8]. In addition to the offset, the change in leg length after THA is considered an important factor for a physiological gait pattern and high patient satisfaction [8,9]. There is controversy in the literature about how hip, knee and more distal joint parameters influence each other [10,11]. There is a lag in understanding the impact of changing hip parameters following THA on the knee. For several decades, the so-called “safe zone”, according to Lewinnek, with an acetabular inclination of 30–50° and an acetabular anteversion of 5–25°, was considered the gold standard for the implantation of the acetabular component [12]. Due to the increasing number of different prosthesis designs and better understanding of the biomechanics of the artificial hip joint, these target values for component placement are now considered in a much more differentiated way [13]. The so-called “combined safe zone” describes a combination of component alignment, component design parameters and the achievable range of motion after THA [14]. In this context, the combination of cup positioning and the alignment of the stem plays a crucial role in the impingement-free range of motion resulting from the combined anteversion of both components [14,15,16,17]. Furthermore, prosthesis design, particularly the use of short-stem prostheses, and stem positioning have been shown to contribute to offset recovery and thus have a direct impact on postoperative outcomes and patient satisfaction [18,19,20].

In the present study, the effects of stem design (short- vs. straight-stem) and stem placement (varus or valgus) on the total leg axis were examined. It was hypothesized that (1) stem design is associated with changes in femoral offset and leg length, (2) stem design is associated with changes in the long leg axis and (3) stem design has an effect on the positioning of the stem in the femur.

## 2. Materials and Methods

### 2.1. Study Design and Setting

This study analyzed data from patients who participated in a previous prospective study at our clinic. The overall aim of this large prospective study was to compare preoperative planning using hipEOS^®^ (EOS imaging, SA, Paris, France) with preoperative planning based on conventional digital radiographs [21]. A further aim was to investigate the relationship between static leg alignment and gait dynamics [22,23,24]. For the present secondary analysis, only data from patients who underwent biplanar radiography with the EOS^®^ (EOS imaging, SA, Paris, France) system in standing position preoperatively as well as 3 and/or 12 months after THA were included. For all patients in the study group, an uncemented, short or straight stem was planned. The stem type was selected based on the best fit during the planning and intraoperative bone quality.

In all short-stem cases, the partial femoral neck preserving short-stem optimys^®^ (Mathys AG, Bettlach, Switzerland) in the lateralized version was used. This implant has the advantage of being a muscle-sparing, minimally invasive implantation technique, and there is a wide range of implant positions to reconstruct the offset and leg length [19]. Three different types of straight-stem prostheses were used, Alloclassic^®^ Zweymüller^®^ Stem, CLS^®^ Spotorno^®^ Hip Stem and the MS-30^®^ Stem, all from Zimmer (Zimmer Biomet, Warsaw, IN, USA) with the principle of a fixation concept in the femoral diaphysis. The choice of stem design for each patient was based on the patient’s condition, such as age, gender and bone quality, and on individual prosthesis planning using the hipEOS^®^ planning tool. The decision was then made for whether a lateralized stem was required to reconstruct the femoral offset. The head length was standardized and planned in size M, but the surgeon decided on the correct head size intraoperatively to ensure adequate muscle tension and no dislocation tendency. This study was conducted from May 2016 to February 2019. 

### 2.2. Patients

Patients who were scheduled for and underwent THA due to unilateral hip OA and were between 30 and 80 years of age were considered for inclusion. As this study was part of a large prospective study including a gait analysis, the exclusion criteria were inflammatory arthritis, orthopedic surgery within the past 6 months, previous lower extremity joint arthroplasty, body mass index (BMI) above 32, chronic or neuromuscular diseases, musculoskeletal conditions involving any other lower extremity joints or low back pain, and inability to walk without walking aids. All patients provided written informed consent prior to participation. The protocol was approved by the local medical ethics committee.

In this study, 30 patients were included. Data from three patients could not be used: in two patients, the preoperative EOS image and, in one patient, the postoperative EOS image could not be reconstructed because of motion artifacts. The characteristics of the 27 included patients are listed in Table 1. 

### 2.3. Radiographic Measurements

Biplanar radiographs in the standing position were taken with the EOS^®^ system (EOS imaging, SA, Paris, France) for all patients preoperatively as well as 3 and/or 12 months postoperatively [25,26]. For each patient, a 3D reconstruction of the lower extremities was provided by EOS imaging from the lateral and anterior images using sterEOS^®^ (EOS imaging, SA, Paris, France) [27,28].

### 2.4. Patient-Reported Outcome Measures (PROMs)

Before and one year after surgery, patients’ function and symptoms were assessed using of the Hip injury and Osteoarthritis Outcome Score (HOOS) [29,30] and the UCLA Activity Scale [31]. The HOOS is a joint-specific self-report questionnaire that is reliable for assessing baseline function and change over time in individuals with hip osteoarthritis. The HOOS questionnaire consists of 40 questions divided into five subscales, and each subscale generates a score ranging from 0 to 100, with 100 being the best possible score. The UCLA Activity Scale consists of 10 activity levels ranging from inactive (level 1) to regular participation in impact sports (level 10).

### 2.5. Data Analysis

Data on the implanted stem were obtained from medical records. A 3D reconstruction of the EOS images was used to obtain leg alignment parameters (Table 2). In addition to the values determined from the 3D EOS image, the position of the femoral stem and the acetabular offset were measured two-dimensionally (by one experienced orthopedic surgeon (BF)) using mediCAD planning software (version 3.50; Hectec, Landshut, Germany).

### 2.6. Statistical Analysis

For all parameters, the deltas between the postoperative and preoperative values were determined. Statistical analysis was performed with BiAS for Windows (version 11.10, Hanns Ackermann, epsilon-Verlag Hochheim Darmstadt). Shapiro–Wilk tests were used to check for a normal distribution. Patients were divided into those who received a short-stem prosthesis and those who received a straight-stem prosthesis. Differences in anthropometrics, and the radiographic measurements between groups were examined with independent sample Students’ *t*-tests, whereas differences in PROMs were examined with Mann–Whitney U tests. A Chi-square test was used to compare the sex distribution between groups, and the effect size Cohen’s d was calculated for the significant differences. The level of significance was set at *p* < 0.05.

## 3. Results

### 3.1. Patients

The patients who received a short-stem prosthesis were significantly younger than the patients who received a straight-stem prosthesis (57.2 ± 11.5 vs. 67.5 ± 6.6 years, *p* = 0.010, d = 1.12). No differences between groups were found for height, weight, body mass index or sex (Table 1).

The implanted short-stem prostheses were all optimys lateral (Mathys AG, Bettlach, Switzerland). Three different straight stems were implanted: Alloclassic Zweymüller, CLS Spotorno and MS-30 (Zimmer Biomet, Warsaw, IN, USA). Ten of the fourteen straight stems had a lateralized offset (9 Alloclassic SLO, 1 MS-30 lateral).

### 3.2. Patient-Reported Outocme Measures (PROMs)

Preoperatively, 25 patients (11 in the short-stem group and 14 in the straight-stem group) completed the questionnaires. Only 23 patients (11 in the short-stem group and 12 in the straight-stem group) completed the questionnaires one year postoperatively. A significant improvement was found for all subscores of HOOS as well as for the UCLA one year postoperatively (*p* < 0.001). There were no differences between the groups, neither before nor one year after THA (Table 3). 

### 3.3. Leg Alignment Parameters

The analysis of the short- and straight-stem groups revealed no significant differences in the preoperative leg alignment parameters (Table 4). 

The preoperative to postoperative changes in the radiological leg alignment parameters of the respective groups are presented in Table 5. The comparison of the short- and straight-stem groups revealed no significant difference in the pre- to postoperative leg length changes (*p* > 0.671). In the present population, the implantation of a short-stem prosthesis resulted in a significantly greater increase in FO offset than did the implantation of a straight-stem prosthesis (11.4 ± 5.9 vs. 4.6 ± 7.4 mm, *p* = 0.014, d = 1.02). In this context, patients with a short-stem prosthesis showed a significantly greater decrease in AO than patients with a straight-stem prosthesis (−5.7 ± 4.3 vs. −2.4 ± 3.8 mm, *p* = 0.042, d = 0.83). 

Compared with the implantation of a straight-stem prosthesis, the implantation of a short-stem prosthesis resulted in a significantly greater varus orientation (HKA) of the leg (−1.4 ± 0.9 vs. −0.4 ± 1.4°, *p* = 0.048, d = 0.80). Further changes were detected in the NSA (*p* = 0.043, d = 0.82): the NSA increased in the short-stem group, but it decreased in the straight-stem group.

The position of the prosthesis within the shaft, determined by the stem–femur diaphysis angle, did not differ between the groups: 3.6 ± 3.1 vs. 2.6 ± 1.9° for the short- and straight-stem groups, respectively (*p* = 0.317). Nevertheless, the short-stem prostheses were positioned more variably but not significantly differently from the straight-stem prostheses.

## 4. Discussion

In this study, the effects of stem design and stem positioning on the offset and total leg axis were examined. It was found that implantation of short-stem prostheses led to a greater increase in FO and varus orientation of the leg compared to the implantation of a straight stem. The short-stem prostheses were positioned more variably but not significantly differently from the straight-stem prostheses.

The PROMs (UCLA and all subscores of HOOS) increased after THA to a level slightly below that of unaffected persons [32]. No differences in outcomes were found between the short-stem group and the straight-stem group. Several studies have found that PROMs are not sensitive enough to show the effect of differences in hip joint reconstruction; however, the results are inconclusive. Bolink et al. [33] found that patients with deficient FO reconstruction after THA had worse WOMAC function scores at 3 and 12 months. Bjordal and Bjorgul [34] compared the Harris Hip Score and HOOS in patients with normal FO and patients with increased FO and found no significant differences. Similarly, Wylde et al. [35] found no differences in WOMAC scores between patients with normal, increased or decreased FO after THA. Comparable results were found for differences in leg length after THA. Mahmood et al. [36] found significantly less improvement in WOMAC scores after THA for patients with leg lengthening compared to patients with leg shortening, but no significant differences when compared to patients with adequate leg length restoration. Zhang et al. [37] found improved Harris Hip Scores for patients with smaller leg length differences after THA surgery. The aforementioned studies and the results of the present study emphasize that in patients with small differences in postoperative outcomes, i.e., within the range of the contralateral side, patient-reported outcomes are not better or worse.

No significant difference in pre- to postoperative leg length changes was found; however, the implantation of a short-stem prosthesis resulted in a significantly greater increase in FO than did the implantation of a straight-stem prosthesis. As the FO increased more, the AO decreased more in the short-stem group. From a biomechanical standpoint, it is reasonable in THA to medially reposition the center of rotation of the acetabulum slightly, as this leads to reduced hip joint forces and consequently less wear on the prosthesis, especially the acetabular inlay [38]. As shown in Table 5, the medialization of the cup was performed in patients and resulted in a reduced AO. To restore the global offset, it is subsequently necessary to increase the FO so that sufficient muscle preloading of the ipsilateral hip abductors can be achieved [39]. The present study suggests that this FO compensation, also with the aim of intra- and postoperative prevention of a dislocation, can be affected by short-stem prostheses. However, the effects on the total leg axis resulting from the femoral offset increase must always be considered to minimize leg varisation and thus the risk of progression of medial gonarthrosis [40,41]. In the present study, due to the increase in FO, the NSA and the HKA increased with the implantation of the short-stem prosthesis. 

The data in the present study showed that short-stem prostheses are inserted more variably than straight-stem prostheses. As the stem geometry is oriented and adapted to the calcar region of the proximal femur, adjusting the femoral neck resection height can directly influence varus or valgus stem positioning [19]. Short-stem prostheses have a wide range of applications for offset reconstruction due to their high adaptability to anatomical conditions, which can be achieved by adjusting femoral neck resection. However, the patient-specific adaptation of femoral neck resection also places special demands on prosthesis planning and on the surgeon’s planning. In the present study, the FO was reconstructed using the lateralized stem model. The use of the standard version of the Optimys stem in a more varus orientation is more common among experienced short-stem surgeons. Thus, even greater differences can be expected in positioning between short- and straight-stem designs.

The stem position did not correlate with the change in the total leg axis. One possible reason could be the overall relatively neutral shaft position in most patients in the examined group and the use of the lateralized short-stem component. Xu et al. suggested that varus positioning in a straight stem has a significant effect on the FO [42]. To avoid unexpected offset changes, it is mandatory to implant the stem component exactly as initially planned, and meticulous attention should be devoted to potential offset changes during the planning process. A varus position of the stem has no effect on the prosthesis stem life, as in the study of Kutzner et al. [18], in which no premature aseptic loosening rates were found.

This study has several limitations. The small sample size (N = 27) may have limited the power of the subgroup analyses and the generalizability of the results. Due to the small sample size, it was decided not to further categorize the patients into smaller subgroups such as coxa vara, coxa valga, or protrusion of the femoral head and neck. A large multicenter study is needed for such an analysis. Due to the final intraoperative decision as to which implant was chosen, two patients received hybrid fixation with a cemented stem after the intraoperative detection of soft bone. Because of the straight-stem design and the small sample size, we included these patients in the straight-stem group. Despite the small number of patients, for the comparison between the stem variants, the post hoc calculated power for the main outcome parameters was acceptable (0.53–0.72). In the study group, the surgeons used short-stem prostheses with lateralized stem designs to achieve better stabilization of the THA joint. With greater experience of surgeons with femoral neck-sparing short-stem implants, more accurate offset reconstructions could be achieved [43].

## 5. Conclusions

In conclusion, this study utilized the EOS system to examine the immediate impact of prosthesis stem design on the FO and the overall leg axis. Within our patient cohort, the implantation of short stems was associated with an increase in offset and varus deviation of the leg axis. The implantation of short stems was more variable and hence requires precise planning to reduce the risk of atypical offset increases and leg axis varus deviations due to the variable insertion options for short-stems. 

## Figures and Tables

**Figure 1:**
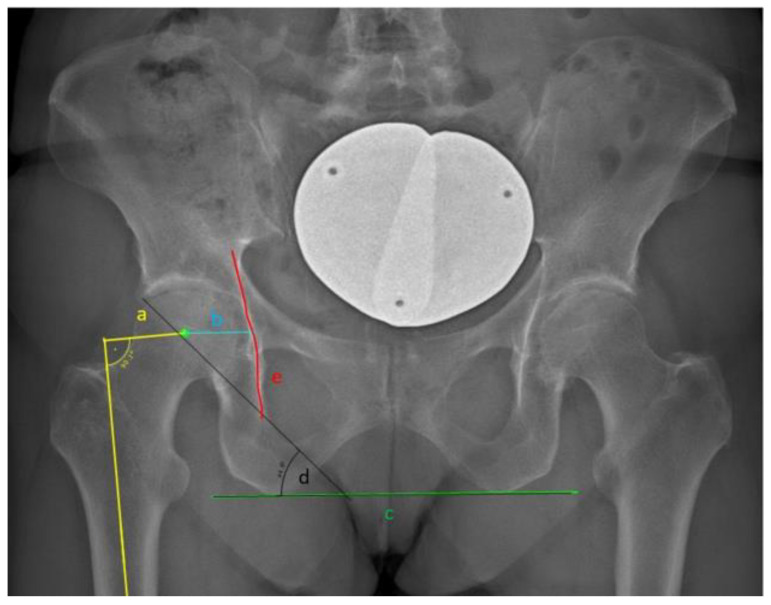
EOS image in the frontal plane showing the pelvis with offset dimensioning. a: Femoral offset; b: Acetabular offset; c: line connecting the most distal points of the tuber ischiadica; d: inclination angle; e: ilioischial line.

**Figure 2:**
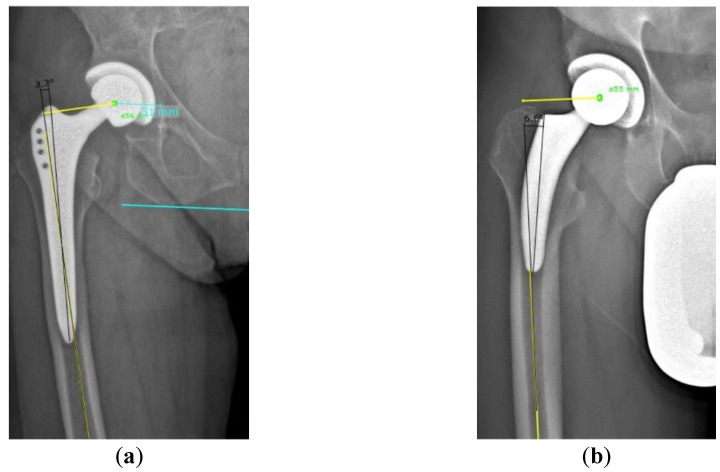
Measurement of the prosthesis positioning: (**a**) for a straight-stem prosthesis; (**b**) for a short-stem prosthesis.

**Table 1:** Anthropometric data of the patients.

	Patients *n* = 27	Short Stem *n* = 13	Straight Stem *n* = 14	*p*-Value
Age (years)	62.5 ± 10.5	57.2 ± 11.5	67.5 ± 6.6	0.010 *
Height (m)	1.70 ± 0.10	1.69 ± 0.10	1.71 ± 0.10	0.719
Weight (kg)	82.1 ± 16.0	84.9 ± 11.3	79.6 ± 19.5	0.385
Body mass index (kgm^−2^)	28.3 ± 4.5	29.7 ± 4.1	26.9 ± 4.6	0.116
Sex (men/women)	12/15	5/8	7/7	0.547

Comparisons between the short-stem and straight-stem groups with corresponding *p*-values (independent-sample *t*-tests/Chi-square test). * Significant difference.

**Table 2:** Definition of measured leg alignment parameters.

Parameter	Definition
Femoral length (cm)	distance between the center of the femoral head and the center of the femoral trochlea.
Tibial length (cm)	distance between the center of the tibial plateau and the center of the distal tibial articular surface.
Functional leg length (cm)	distance between the center of the femoral head and the center of the distal tibial articular surface.
Anatomical leg length (cm)	sum of the femoral length and tibial length.
Femoral offset (mm)	FO: the distance from the center of rotation of the femoral head to a line bisecting the long axis of the femur.
Acetabular offset (mm)	AO: measured as the distance between the center of the femoral head and the ilioischial line (Figure 1).
Hip-Knee-Shaft-angle (°)	HKS: the frontal plane angle measured between the mechanical femoral axis and an axis running from the center of the trochlea to the center of the distal diaphysis.
Neck-Shaft-Angle (°)	NSA: the angle measured between the femoral diaphyseal axis and the axis going from the center of the femoral head through the femoral neck.
Hip-Knee-Angle (°)	HKA: represents the varus/valgus configuration of the knee. The HKA was defined as the angle in the frontal femoral plane between the mechanical axes of the femur and the tibia (the line from the center of the tibial plateau to the center of the distal articular surface of the tibia). A value greater than 0 ° equals a valgus alignment, and a value smaller than 0 ° equals a varus alignment.
Prosthesis stem-femur diaphysis angle (°)	angle between the anatomical femoral shaft axis and the longitudinal axis of the prosthesis stem. The anatomical femoral shaft axis was determined by connecting the centers of the two shaft diameters. The prosthesis stem axis was determined depending on the type of prosthesis. For the straight-stem prosthesis, the prosthesis axis was determined by connecting the proximal and distal points of the prosthesis (Figure 2a). For the short-stem, the axis was defined as the line connecting the distal end of the prosthesis and the lateral proximal shoulder of the prosthesis (Figure 2b).

**Table 3:** Patient-reported outcome measures.

		Short Stem	Straight Stem	*p*-Value
HOOS Pain	pre	40.0 (17.5–60)	45.0 (12.5–60)	0.890
	post	96.3 (65.0–100.0)	97.5 (47.5–100.0)	0.716
HOOS Symptoms	pre	40.0 (15.0–70.0)	42.5 (5.0–75.0)	0.978
	post	95.0 (60.0–100.0)	90.0 (40.0–100.0)	0.682
HOOS ADL	pre	43.3 (11.8–64.3)	45.5 (9.4–66.2)	0.784
	post	94.1 (69.1–100.0)	94.1 (59.4–100.0)	0.853
HOOS Sport/Recreation	pre	21.9 (0–75.0)	25.0 (0–43.8)	0.704
	post	93.8 (50.0–100.0)	81.3 (37.5–100.0)	0.281
HOOS QL	pre	18.8 (0–56.3)	18.8 (0–43.8)	0.883
	post	71.9 (37.5–100.0)	68.8 (25.0–100.0)	0.745
UCLA	pre	5.5 (3.0–9.0)	4.5 (3.0–10.0)	0.958
	post	7.0 (5.0–10.0)	9.0 (5.0–10.0)	0.268

Comparison between the short-stem and straight-stem groups with corresponding *p*-values (Mann–Whitney U tests). Abbreviations: ADL: activities of daily living; QL: quality of life.

**Table 4:** Preoperative leg alignment parameters of the patients.

	Short Stem *n* = 13	Straight Stem *n* = 14	*p*-Value
Femoral length (cm)	43.1 ± 3.1	43.3 ± 3.0	0.862
Tibial length (cm)	37.1 ± 3.1	37.5 ± 2.7	0.671
Functional leg length (cm)	80.5 ± 6.3	80.9 ± 5.5	0.835
Anatomical leg length (cm)	80.1 ± 6.2	80.8 ± 5.7	0.764
Femoral offset (mm)	38.0 ± 5.7	41.9 ± 9.2	0.207
Acetabular offset (mm)	34.2 ± 5.0	32.9 ± 5.5	0.524
Hip–knee–shaft angle (°)	4.6 ± 1.5	4.9 ± 1.2	0.526
Neck–shaft angle (°)	129.3 ± 6.0	126.0 ± 6.5	0.179
Hip–knee angle (°)	−1.0 ± 2.7	−1.0 ± 3.3	0.990

Comparison between the short-stem and straight-stem groups with corresponding *p*-values (independent-sample *t*-tests).

**Table 5:** Changes in leg alignment parameters between the preoperative and postoperative values.

	Patients *n* = 27	Short Stem *n* = 13	Straight Stem *n* = 14	*p*-Value
Δ Femoral length (cm)	0.7 ± 0.5	0.8 ± 0.4	0.7 ± 0.6	0.811
Δ Tibial length (cm)	0.0 ± 0.1	0.0 ± 0.1	0.0 ± 0.1	0.479
Δ Functional leg length (cm)	0.9 ± 0.6	0.8 ± 0.4	0.9 ± 0.7	0.920
Δ Anatomical leg length (cm)	0.7 ± 05	0.8 ± 0.4	0.7 ± 0.6	0.672
Δ Femoral offset (mm)	7.9 ± 7.5	11.4 ± 5.9	4.6 ± 7.4	0.014 *
Δ Acetabular offset (mm)	−4.0 ± 4.3	−5.7 ± 4.3	−2.4 ± 3.8	0.042 *
Δ Hip–knee–shaft angle (°)	1.2 ± 0.8	1.3 ± 0.6	1.0 ± 1.0	0.444
Δ Neck–shaft angle (°)	−0.6 ± 6.4	2.0 ± 5.6	−3.0 ± 6.4	0.043 *
Δ Hip–knee angle (°)	−0.9 ± 1.3	−1.4 ± 0.9	−0.4 ± 1.4	0.048 *

Comparison between the short-stem and straight-stem groups with corresponding *p*-values (independent-sample *t*-tests). * Significant difference.

## Data Availability

De-identified subject data have been deposited in the Goethe University Data repository (https://doi.org/10.25716/gude.17z0-08d0).
